# Fighting Post-COVID and ME/CFS – development of curative therapies

**DOI:** 10.3389/fmed.2023.1194754

**Published:** 2023-06-15

**Authors:** Carmen Scheibenbogen, Judith Theresia Bellmann-Strobl, Cornelia Heindrich, Kirsten Wittke, Elisa Stein, Christiana Franke, Harald Prüss, Hannah Preßler, Marie-Luise Machule, Heinrich Audebert, Carsten Finke, Hanna Gwendolyn Zimmermann, Birgit Sawitzki, Christian Meisel, Markus Toelle, Anne Krueger, Anna C. Aschenbrenner, Joachim L. Schultze, Marc D. Beyer, Markus Ralser, Michael Mülleder, Leif Erik Sander, Frank Konietschke, Friedemann Paul, Silvia Stojanov, Lisa Bruckert, Dennis M. Hedderich, Franziska Knolle, Gabriela Riemekasten, Maria J. G. T. Vehreschild, Oliver A. Cornely, Uta Behrends, Susen Burock

**Affiliations:** ^1^Institute of Medical Immunology, Charité - Universitätsmedizin Berlin, corporate member of Freie Universität Berlin and Humboldt Universität zu Berlin, Berlin, Germany; ^2^Experimental and Clinical Research Center, a cooperation between the Max Delbrück Center for Molecular Medicine in the Helmholtz Association and Charité Universitätsmedizin Berlin, Berlin, Germany; ^3^NeuroCure Clinical Research Center, Charité – Universitätsmedizin Berlin, corporate member of Freie Universität Berlin and Humboldt-Universität zu Berlin, Berlin, Germany; ^4^Max Delbrück Center for Molecular Medicine in the Helmholtz Association (MDC), Berlin, Germany; ^5^Department of Neurology, Charité - Universitätsmedizin Berlin, corporate member of Freie Universität Berlin and Humboldt Universität zu Berlin, Berlin, Germany; ^6^Deutsches Zentrum für Neurodegenerative Erkrankungen (DZNE), Berlin, Germany; ^7^Berlin Institute of Health at Charité – Universitätsmedizin Berlin, Center of Immunomics, Berlin, Germany; ^8^Department of Immunology, Labor Berlin - Charité Vivantes GmbH, Berlin, Germany; ^9^Department of Nephrology, Charité - Universitätsmedizin Berlin, corporate member of Freie Universität Berlin and Humboldt Universität zu Berlin, Berlin, Germany; ^10^Systems Medicine, Deutsches Zentrum für Neurodegenerative Erkrankungen (DZNE), Bonn, Germany; ^11^PRECISE Platform for Single Cell Genomics and Epigenomics, DZNE and University of Bonn, Bonn, Germany; ^12^Genomics and Immunoregulation, Life and Medical Sciences Institute, University of Bonn, Bonn, Germany; ^13^Institute of Biochemistry, Charité – Universitätsmedizin Berlin, corporate member of Freie Universität Berlin and Humboldt-Universität zu Berlin, Berlin, Germany; ^14^The Wellcome Centre for Human Genetics, Nuffield Department of Medicine, University of Oxford, Oxford, United Kingdom; ^15^Department of Infectious Diseases and Respiratory Medicine, Charité–Universitätsmedizin Berlin, corporate member of Freie Universität Berlin and Humboldt-Universität zu Berlin, Berlin, Germany; ^16^Institute of Biochemistry, Charité – Universitätsmedizin Berlin, corporate member of Freie Universität Berlin and Humboldt-Universität zu Berlin, Berlin, Germany; ^17^Childrens’ Hospital, School of Medicine, Technical University of Munich, Munich, Germany; ^18^Clinical Trial Office, Charité - Universitätsmedizin Berlin, corporate member of Freie Universität Berlin and Humboldt Universität zu Berlin, Berlin, Germany; ^19^Department of Neuroradiology, School of Medicine, Technical University of Munich, Munich, Germany; ^20^Department of Rheumatology, University Medical Center Schleswig-Holstein Campus Lübeck, Lübeck, Germany; ^21^Department of Internal Medicine, Infectious Diseases, University Hospital Frankfurt, Goethe University Frankfurt, Frankfurt am Main, Germany; ^22^Department of Internal Medicine, University Hospital Cologne, Cologne, Germany; ^23^German Centre for Infection Research (DZIF), Partner Site Bonn-Cologne, Germany; ^24^University of Cologne, Faculty of Medicine, Institute of Translational Research, Cologne Excellence Cluster on Cellular Stress Responses in Aging-Associated Diseases (CECAD), Cologne, Germany; ^25^German Center for Infection Research (DZIF), Berlin, Germany; ^26^AGV Research Unit Gene Vectors, Helmholtz Center Munich (HMGU), Munich, Germany

**Keywords:** COVID-19, post-COVID, ME/CFS, inflammation, endothelial dysfunction, autoantibodies, clinical trials

## Abstract

The sequela of COVID-19 include a broad spectrum of symptoms that fall under the umbrella term post-COVID-19 condition or syndrome (PCS). Immune dysregulation, autoimmunity, endothelial dysfunction, viral persistence, and viral reactivation have been identified as potential mechanisms. However, there is heterogeneity in expression of biomarkers, and it is unknown yet whether these distinguish different clinical subgroups of PCS. There is an overlap of symptoms and pathomechanisms of PCS with postinfectious myalgic encephalomyelitis/chronic fatigue syndrome (ME/CFS). No curative therapies are available for ME/CFS or PCS. The mechanisms identified so far provide targets for therapeutic interventions. To accelerate the development of therapies, we propose evaluating drugs targeting different mechanisms in clinical trial networks using harmonized diagnostic and outcome criteria and subgrouping patients based on a thorough clinical profiling including a comprehensive diagnostic and biomarker phenotyping.

## Introduction

COVID-19 frequently results in persistent debilitating symptoms lasting longer than 3 months, referred to as post-COVID-19 syndrome (PCS). Based on large epidemiological studies, approximately 10% of adults who had a positive SARS-CoV-2 PCR suffer from persisting symptoms beyond 3 months (1). Vaccination confers partial protection against PCS (2). In the majority of adult PCS patients, severity of symptoms persists or even increases after 12 months (3). Predominantly, healthy young and middle-aged adults with female preponderance are affected. Less data is available for children and adolescents, indicating a lower prevalence and severity (4, 5).

The clinical presentation is complex with various clinical phenotypes and most likely different mechanisms (6, 7). In most younger patients, there is no evidence for organ damage, and the majority has a symptom cluster with predominance of fatigue, exertion intolerance, cognitive impairment, orthostatic intolerance, and autonomous dysfunction (8).

Postinfectious syndromes have been described for more than a century and can be triggered by various infections (9). There is now clear evidence that a subset of PCS complies with standard case definitions of myalgic encephalomyelitis/chronic fatigue syndrome (ME/CFS) (10, 11). We will refer to such cases as post-COVID (PC) ME/CFS. Post-infectious ME/CFS (ICD-10 code G93.3) is a complex and severely disabling disease with no approved treatment and therefore, a very high and so far unmet medical need (12). Key symptoms are central and muscle fatigue, exertion intolerance with post-exertional malaise (PEM), cognitive impairment, orthostatic intolerance, headache, and muscle pain - hence a large overlap of symptoms with PCS. ME/CFS had an estimated prevalence of 0.3–0.8% before COVID-19, including children and adolescents (12). The prevalence of PCS that fits ME/CFS diagnostic criteria will likely be substantial, and poses a major problem for health care and society. A recent study from Germany that analyzed health insurance data from nearly 30 million individuals showed an annual incidence rate in 2020 of 6 versus 2/1000 in adults with and without prior COVID-19, respectively (13). So far, there is no proven effective therapy for PCS or ME/CFS (14).

## Pathomechanisms of pcs and Me/Cfs

There is accumulating evidence from large well-performed studies that immune activation and dysregulation with inflammation and alteration of immune cells are frequently found in PCS. Various autoantibodies have been described to be triggered by COVID-19 and to be associated with the development of PCS. Correlations of both, soluble markers of inflammation and autoantibodies, including antinuclear antibodies (ANA), neurological and G protein-coupled receptor (GPCR) antibodies with symptom severity were found (15–17). Endothelitis is common in acute COVID-19, and can persist in PCS with endothelial dysfunction and various biomarkers of endothelial inflammation, microclots and hypoperfusion shown (7, 18). There is evidence for viral persistence with detection of spike and nucleoprotein in serum in a subset of PCS (7, 19). However, there is a broad heterogeneity in expression of biomarkers, and it is unknown yet whether we face distinct subgroups of PCS or overlapping mechanisms. Although there are correlations of some biomarkers with symptom severity, it is not possible to delineate clinical phenotypes from biomarker profiles yet. Similarly, there is evidence that immune dysregulation and autoantibodies play a key role in ME/CFS, including the high frequency of autoimmune diseases among first-degree family members, associations with autoimmunity-related gene variants and MHC alleles, skewed B cell receptor genes, and association of symptom severity with GPCR antibodies (15, 20–22). Further, there is ample evidence for endothelial dysfunction affecting both medium arteries, assessed by flow-mediated dilation, and capillaries assessed by post occlusive reactive hyperemia (20, 23). Many reports have found altered cytokine levels and their correlation with severity in ME/CFS, though many are inconsistent with each other (24–26). Of interest, reactivation of Epstein–Barr virus (EBV) during COVID-19 frequently occurs and is a risk factor to develop PCS (6, 7). In a subset of individuals, infectious mononucleosis precedes ME/CFS (27).

## Drugs of interest to study in pcs and Me/Cfs

Conceptually, selection criteria for therapeutic strategies should be based on potential mechanisms, defined by specific biomarkers. There are numerous drugs already licensed for other indications that target mechanisms identified in PCS and/or ME/CFS. Repurposing of such drugs may offer faster clinical approval.

In PCS, several interventional randomized controlled trials (RCT) have been initiated for targeted drug therapy worldwide (Table 1). This includes anti-inflammatory drugs, including corticosteroids, loratadine, montelukast, atorvastatin, baricitinib and phosphodiesterase inhibitors. Treatment studies depleting autoantibodies have been started with plasma exchange and immunoadsorption. The first specific drug in a clinical trial is the neonatal Fc receptor inhibitor efgartigimod, which enhances IgG degradation and was recently licensed for therapy in myasthenia gravis (28). Another study was initiated with the aptamer BC007, which has shown safety and GPCR antibody neutralizing capacity in a phase I trial (29). Antivirals include targeting of potential residual SARS-CoV-2 as well as a monoclonal antibody against a reactivated endogenous retrovirus. Further several neuromodulators are studied in RCT, including vortioxetine, an antidepressant with established pro-cognitive properties, lithium with anti-depressive and anti-inflammatory properties, fampridine, a potassium channel-blocking agent linked to working memory and approved for multiple sclerosis, and dexamphetamine with first evidence for efficacy in ME/CFS (30). Targeting endothelial dysfunction and hypoperfusion holds promise in both, PCS and ME/CFS, and a phase II RCT with the guanylate cyclase inhibitor vericiguat already licensed in heart failure has started in PCS and PC ME/CFS (31). Hyperbaric oxygen therapy (HBOT) was already shown to improve neurocognitive function in a phase II RCT in PCS, and is currently studied in another RCT (32).

**Table 1:** Randomized controlled trials in PCS registered in clinical trial platforms^*^.

Trial	Interventions under comparison	PC/subtype	Country	Current state	No. of subjects	NCT
Immunomodulatory						
Phase 2 RCT	IgG vs. methylprednisolone vs. saline	PC neurological	USA	Recruiting	45	NCT05350774
Phase 3 RCT (open)	Atorvastatin vs. standard care	LC neurocognitive	Australia	Recruiting	400	NCT04904536
Phase 2 RCT	Plasma Exchange Therapy vs. sham	PC	Spain	not yet recruiting	50	NCT05445674
Phase 2 RCT	Immunoadsorption vs. sham	PC ME/CFS (CCC) and autoantibodies	Germany	Not yet recruiting	66	NCT05710770
Phase 3 RCT	Montelukast vs. placebo	LC respiratory	Spain	Recruiting	284	NCT04695704
Phase 2 RCT	Ampligen vs. saline	PC ME/CFS (CDC)		Not yet recruiting	80	NCT05592418
Phase 3 RCT	Prednisolone (low dose) vs. placebo Vitamin B1/6/12 vs. placebo	PC	Germany	Recruiting	340	NCT05638633
Phase 2 RCT	Efgartigimod vs. placebo	PC POTS	USA	Recruiting	42	NCT05633407
Phase 2/3 RCT adaptive	Ibudilast vs. Pentoxifylline vs. placebo	PC	Canada	Not yet recruiting	1,000	NCT05513560
Phase 2 RCT	Baricitinib vs. placebo	PC cognitive	USA	Not yet recruiting	30	NCT05858515
Phase 2 RCT	BC007 aptamer vs. placebo	LC fatigue	Germany/Europe	Not yet recruiting	114	EudraCT2022-003452-14
Phase 4 RCT	Loratidine vs. placebo	LC	India	Not yet recruiting	64	CTRI/2022/07/043679
Vascular						
Phase 2 RCT	Hyperbaric oxygen therapy vs. sham	PC or LC	Sweden	Recruiting	80	NCT04842448
Phase 2 RCT	Vericiguat vs. placebo	PC ME/CFS (CCC or IOM) and endothelial dysfunction	Germany	Not yet recruiting	104	NCT05697640
Antiviral						
Phase 2 RCT	Paxlovid vs. Ritonavir vs. placebo	PC	USA	Recruiting	200	NCT05576662
Phase 2 RCT	Temelimab vs. placebo	PC neuropsychiatric	Switzerland	Recruiting	200	NCT05497089
Phase 3 RCT	Meplazumab (anti-CD147) vs. placebo	PC (at least one symptom)	China	Not yet recruiting	144	NCT05813587
Neuro-modulators						
Phase 4 RCT (open)	Dextroamphetamine vs. app	PC cognitive	USA	Recruiting	120	NCT05597722
Phase 2 RCT	Low-dose Naltrexone (LDN) vs. placebo	PC ME/CFS	Canada	Not yet recruiting	160	NCT05430152
Phase 2 RCT	Lithium vs. placebo	LC fatigue and/or brain fog	USA	Recruiting	50	NCT05618587
Phase 2 RCT	Vortioxetine vs. placebo	PC cognitive	Canada	Complete	200	NCT05047952
Phase 2 RCT	Fampridine vs. placebo	PC cognitive	Switzerland	Recruiting	44	NCT05274477
Phase 2 RCT	Ketamine vs. placebo	PC depressive	USA	Recruiting	12	NCT05690503

^*^ClinicalTrials.gov, https://clinicaltrials.gov/; EU Clinical Trials Register https://www.clinicaltrialsregister.eu/; International Clinical Trials Registry Platform (ICTRP), https://www.who.int/clinical-trials-registry-platform (date 22.5.2023); RCT, randomized controlled trial; PC: Post-COVID-19 Condition or Syndrome, LC: Long Covid; NCT: National Clinical Trials Number = ClinicalTrials.gov Identifier.

In ME/CFS, there has been little interest of pharmaceutical companies in clinical trials for decades, presumably due to the complexity of the disease, conflicting concepts of etiology and paucity of research on pathomechanisms. There are now 2 trials conducted in PC ME/CFS including rintatolimod, a TLR-3 agonist. This is one of the few drugs, which has been studied in a phase III trial in ME/CFS showing evidence for efficacy in patients with shorter disease duration (33). Inclusion criteria are, however, the Fukuda criteria not requiring PEM. Further low dose naltrexone with evidence for efficacy from case reports in ME/CFS and recently also in an open trial in PCS will be studied in a RCT phase 2 trial (34, 35). For non-PC ME/CFS only one interventional RCT pharmacological trial with N-acetylcysteine could be found (NCT04542161). However, there is reasonable hope that some of the drugs that are effective in LC can also be used in ME/CFS.

Further drugs of interest to study in PCS and ME/CFS are listed in Table 2. A novel approach to alleviate inflammation is to target kinases that regulate inflammatory mediators like Janus kinase (JAK) inhibitors and several others (36, 37). Further certain drugs exert, besides their licensed indication, anti-inflammatory effects like metformin or certain antibiotics with evidence for efficacy in acute COVID or from non-controlled trials in ME/CFS like minocycline (36, 39, 40). Further, there is first data that H1 and H2 antihistamines can have beneficial effects (38). There are numerous drugs to target autoantibody-producing B cells including monoclonal antibodies and more recently Bruton tyrosinkinase (BTK) inhibitors. Rituximab has been studied in ME/CFS in several phase II and one phase III study with inconclusive results (49, 50). Newer and more effective antibodies targeting CD20, CD19 or CD38 depleting both, B cells and/or plasma cells, are thus promising candidates (41). There are further groups of drugs targeting endothelial dysfunction *via* PDE5, β2/3 adrenergic or acetylcholine receptors (43–45). A small study was already performed with the PDE5 inhibitor sildenafil in ME/CFS showing a significant improvement of fatigue in 5 treated patients compared to 6 receiving placebo (NCT00598585). There is also evidence from various small trials in ME/CFS that the neuromodulators low dose aripiprazole and methylphenidate can have efficacy (46, 47). Also guanfacine was described in a case series to ameliorate symptoms in PCS (48). PCS and ME/CFS patients frequently suffer from dysautonomia and postural tachycardia syndrome (POTS). There are several pharmacological treatment options from small clinical trials, but no licensed drugs are available. Similarly, for other key symptoms of ME/CFS and PCS, sleep disturbances and post exertional malaise there is no evidence for medications from clinical trials.

**Table 2:** Further drugs of interest in PCS and ME/CFS.

Target	Drugs	References
Inflammation	Kinase inhibitors	Ref. (36, 37)
	Antihistamines (H1 + H2)	Ref. (38)
	Minocycline	Ref. (39)
	Metformin	Ref. (40)
Autoantibodies	CD20 monoclonal antibodies targeting B-cells	Ref. (20)
	CD19 monoclonal antibodies targeting B-cells	Ref. (41)
	BTK inhibitor	Ref. (42)
Vascular	Pyridostigmine	Ref. (43)
	β2/3 receptor agonists	Ref. (44)
	PDE5 inhibitor	NCT00598585
	Nicotine	Ref. (45)
Neuromodulation	Low dose aripiprazole	Ref. (46)
	Methylphenidate	Ref. (47)
	Guanfacine	Ref. (48)

## Concept for clinical trial networks

Due to the complexity of PCS and ME/CFS harmonization of diagnostic and inclusion and outcome criteria for clinical trials would be desirable. So far, many clinical trials do not specify PCS subgroups or clinical phenotypes. In several ongoing trials Long COVID (LC) is mentioned as inclusion criterium, which is poorly defined as persistent symptoms for more than 4 weeks. For ME/CFS, various diagnostic criteria exist and only stricter criteria requiring the cardinal symptom PEM should be used for clinical trials (12). Clinical trial platforms or networks would allow proof-of-concept clinical trials with various drugs in a harmonized manner using similar diagnostic criteria, evaluation tools, clinical outcome criteria and pre-enrolment phenotyping of patients to categorize them according to potential underlying mechanisms. Further clinical trial networks allow to rapidly recruit larger sample size when moving from phase II to phase III trials or to recruit ME/CFS patients triggered by another infection, e.g., EBV. Due to the diversity of pathological mechanisms, clinical trials should be accompanied by comprehensive biomarker analyses, focusing on both, classical biomarkers as well as compound biomarkers, and biomarker signatures that become increasingly accessible. Besides achieving further insights into the mechanisms and into drug efficacy, such approaches can lead to the development of companion diagnostics for consecutive trials. Specific diagnostic assessments including advanced structural and functional magnetic resonance imaging (MRI), neurocognitive testing, autonomic testing, and vascular imaging should be implemented to visualize key clinical and functional abnormalities of PCS (18, 51–53).

Based on the concept outlined above, a German consortium was recently established, the National Clinical Study Group (NKSG) for PCS and ME/CFS. The interdisciplinary team includes clinical experts from neurology, neuroimmunology, clinical immunology, rheumatology, cardiology, pediatrics, psychiatry, neuropsychology, neuroradiology, and infection medicine, with specific expertise in diagnosing and treating patients with PCS and ME/CFS, as well as experts in human immunology, molecular medicine, biochemistry, data sciences, bioinformatics, and artificial intelligence (AI), with long-standing expertise in biomedical research. Patient inclusion criteria refer to defined clinical phenotypes, objective clinical measures, and potential biomarkers. Patients with PCS and/or ME/CFS are being diagnosed according to standard diagnostic criteria as published for PCS by the WHO and for ME/CFS by the Canadian Consensus Criteria (54). In addition to quantification of symptoms and functional impairment by specific questionnaires and patient reported outcome measures (PROMs), neurocognitive and autonomic testing, multimodal MRI of the brain, as well as assessment of physical fatigue and endothelial dysfunction will be performed before and after treatment (18, 51–53). Further we offer regular education and support in diagnostic assessment of ME/CFS.

Regulatory requirements make investigator-initiated clinical trials challenging. For clinical trial management, a clinical trial office (CTO) platform aids in protocol preparation and is in charge of all regulatory and data safety affairs, trial submission, monitoring, data management, and biostatistical support. Harmonized clinical study documents including protocols, diagnostic criteria, and clinical outcome parameters are provided for all studies to allow rapid preparation of clinical trials and comparison of outcomes among the various trials. Measures for quality assurance include recruitment of patients from specialized university institutions and from observational studies, the use of standardized diagnostic criteria, and the collection of a harmonized set of data in a secure common database. This approach will allow to perform excellent systematic and comprehensive analyses, and to compare the results across all trials.

Clinical trials will be accompanied by a comprehensive biomarker program to understand pathomechanisms of relevance for drug efficacy and to identify companion diagnostics. The biomarker platform will provide comprehensive phenotyping for all trials including the analyses of soluble markers for inflammation and endothelial dysfunction, autoantibodies, immune cell phenotyping, viral persistence, and reactivation, as well as high-resolution approaches such as single-cell RNA sequencing (scRNA-seq) and proteomics (15, 55–57). Special attention needs to be given to the observation that single biomarkers often fail to capture the properties of complex diseases. New proteomic techniques allow to measure signatures in human serum und plasma at low costs, and can rapidly be translated into panel assays that suit routine testing (56, 57). High-resolution scRNA-seq can assess all immune cells and deviations of their molecular programs in parallel, allowing unravelling alterations in subpopulations unamenable by routine diagnostics as well as the development of novel signatures for disease and treatment outcome (55, 58). To assure quality of biomaterial and comparability of laboratory results, all blood samples will be collected in a harmonized manner, and then processed and stored according to standard operating procedures (SOPs) at local biobanks.

A diagnostic platform will perform structural and functional MRI studies and vascular diagnostics before and after interventions, including assessments of endothelial function and perfusion using non-invasive detection and measurement of endothelial dysfunction *via* Endo-PAT™, optical coherence tomography angiography (OCT-A), and arterial spin labeling (ASL) MRI (18, 59). Diagnostic assessments will be performed using harmonized protocols that have been previously established within the German National Pandemic Cohort Network (NAPKON) (60).

Links between clinical, diagnostic, and biomarker data will be established *via* bioinformatics, statistics, machine learning, and AI with the overarching goal to identify subgroups responding to the different therapeutic strategies, to further elucidate the pathogenesis of PCS and ME/CFS, and to identify diagnostic and prognostic biomarkers.

The first proof-of-concept trials (in phase II settings) performed are hypothesis-driven with a rationale based on clinical phenotypes and existing biomarkers. Repurposing of drugs will guarantee rapid trial initiation and drug availability. To assess the role of autoantibodies, a proof-of-concept trial with repetitive immunoadsorption in PC ME/CFS (NCT05629988) as well as a randomized controlled trial (RCT) with a sham-apheresis in postinfectious and PC ME/CFS with autoantibodies will be performed (NCT05710770) (61). Patients responding to immunoadsorption but relapsing will be treated in a consecutive trial with a B cell depleting monoclonal antibody. To treat endothelial dysfunction and hypoperfusion in PCS and PC ME/CFS, a phase II trial with the sGC stimulator vericiguat has been initiated (NCT05697640). Positive effects of prednisolone treatment in PCS with neurological symptoms have been suggested in case series (62). A RCT with high dose prednisolone will therefore be performed in PCS with predominant neurocognitive impairment, in which inflammation and autoantibodies targeting brain epitopes are common (17). Hyperbaric oxygen therapy was already shown to improve fatigue and cognitive impairment in a sham-controlled trial in long COVID patients (32). The efficacy of HBOT in patients with ME/CFS with moderate to severe cognitive impairment will be studied, too.

Based on results of these phase II trials, drugs and medical devices will be identified to be further evaluated in phase III trials together with the NAPKON-TIP (National Pandemic Cohort Network – Therapeutic Intervention Platform) supported by the German Network University Medicine (NUM) and international partners. The patient organizations Long Covid Deutschland and Deutsche Gesellschaft für ME/CFS are included and participate in the conception and conduction of all clinical trials as well as in the translation of the biomedical research results. Collaboration with the pharmaceutical industry is desired for fast access to drugs, financial support, achieving rapid licensing, and integrating further drugs to be developed.

## Conclusion

Our concept of a multipronged clinical trial platform approach addresses the complexity and heterogeneity of PCS and ME/CFS, enabling to test numerous drugs in clinical trials in a harmonized manner accompanied by comprehensive mechanistic studies. Such an approach will pave the way for more rapid development of drugs for PCS and ME/CFS to find therapeutic solutions for specific subgroups and finally all patients. Further, it will allow the development and identification of precise diagnostic, prognostic and companion biomarkers ultimately leading to targeted and individualized therapies combatting the different disease mechanisms. Finally, the identification of biomarkers predicting response to treatment provides strong evidence for causative pathomechanisms.

## Author’s note

Further members of the NKSG Study Group are: Fatma Amari, Christine Appelt, Silvia Augustin, Sandra Bauer, Janina Behrends, Fabian Boesl, Benno Bremer, Isabel Bünger, Adeline Dehlinger, Vadim Farztdinov, Manuela Fiedler, Helma Freitag, Anja Freiwald, Anna Hausruckinger, Tim Hartung, Johanna Herzog, Uta Hoppmann, Claudia Kedor, Kristin Kräker, Stephan Krohn, Joseph Kuchling, Carla Leutloff, Lucie Yuanting Li, Philippe Manceau, Maron Mantwill, Kirstin Mittelstraß, Astrid Nümann, Vanessa Raeder, Lukas Reeß, Valentin Riedl, Hadi Salih, Franziska Sotzny, Silvia Thiel, Friederike Ufer, Katrin Vogt, Katharina Wurdack, Claus Zimmer.

## Data availability statement

The original contributions presented in the study are included in the article/supplementary material, further inquiries can be directed to the corresponding author.

## Author contributions

CS, SB, and UB developed the concept of the studies. BS, CaF, HPru, ChF, and JB-S gave important input to the study concepts. CS was the guarantor, wrote the original draft of the paper. CS, UB, BS, CaF, HA, JBS, CM, ACA, JLS, FP, MR, SS, DH, and CH reviewed and edited the paper. CH attested that all listed authors meet authorship criteria and that no others meeting the criteria have been omitted. All authors contributed to the article and approved the submitted version.

## Funding

The clinical trial platform is funded by the Bundesministerium für Bildung und Forschung (German Ministry of Education and Research), Grant 01EP2201.

## Conflict of interest

The Charité Universitaetsmedizin Berlin holds a patent for the use of vericiguat in Post-COVID Syndrome. CS, JB-S, CH, KW, ES, CaF, HPru, HPre, M-LM, HA, ChF, HZ, BS, CM, MT, AK, MR, MM, LS, FKo, FP, LB, and SB are employed at Charité Universitaetsmedizin Berlin.

CM was employed by Labor Berlin - Charité Vivantes GmbH.

The remaining authors declare that the research was conducted in the absence of any comercial or financial relationships that could be construed as a potential conflict of interest.

The Handling Editor NS declared a past collaboration with the Author CS.

## Publisher’s note

All claims expressed in this article are solely those of the authors and do not necessarily represent those of their affiliated organizations, or those of the publisher, the editors and the reviewers. Any product that may be evaluated in this article, or claim that may be made by its manufacturer, is not guaranteed or endorsed by the publisher.

## Figures and Tables

**Table:** 

AI	artificial intelligence
ANA	antinuclear antibodies
ASL	arterial spin labeling
BTK	Bruton tyrosinkinase
CTO	Clinical trial office
EBV	Epstein–Barr virus
Endo-PAT™	Brand name
GPCR	G protein-coupled receptor
HBOT	Hyperbaric oxygen therapy
JAK	Janus kinase
LC	Long COVID
ME/CFS	myalgic encephalomyelitis/chronic fatigue syndrome
MHC	major histocompatibility complex
MRI	magnetic resonance imaging
NAPKON	National Pandemic Cohort Network
NAPKON-TIP	National Pandemic Cohort Network – Therapeutic Intervention Platform
NCT	National Clinical Trials Number = ClinicalTrials.gov Identifier
NKSG	Nationale Klinische Studien Gruppe = National Clinical Study Group
NUM	Network University Medicine
OCT-A	optical coherence tomography angiography
PC	Post COVID
PCS	post-COVID-19 syndrome
PDE5	phosphodiesterase type 5
PEM	post-exertional malaise
POTS	postural tachycardia syndrome
RCT	randomized controlled trials
RNA	Ribonucleic acid
scRNA-seq	single-cell RNA sequencing
sGC	Soluble guanylate cyclase
SOP	standard operating procedures
TLR-3	toll-like receptors 3
WHO	World Health Organisation
